# Prevalence and Risk Factors for Adult Cataract in the Jingan District of Shanghai

**DOI:** 10.1155/2022/7547043

**Published:** 2022-08-31

**Authors:** Yingying Hong, Yang Sun, Xiaofang Ye, Yi Lu, Jianjiang Xu, Jianming Xu, Yinghong Ji

**Affiliations:** ^1^Eye Institute and Department of Ophthalmology, Eye & ENT Hospital, Fudan University, Shanghai 200031, China; ^2^NHC Key Laboratory of Myopia (Fudan University), Key Laboratory of Myopia, Chinese Academy of Medical Sciences, Shanghai 200031, China; ^3^Shanghai Key Laboratory of Visual Impairment and Restoration, Shanghai 200031, China; ^4^Shanghai Key Laboratory of Meteorology and Health, Shanghai Meteorological Service, Shanghai 200030, China

## Abstract

**Purpose:**

We report the prevalence of age-related cataract (ARC) in the Jingan district of Shanghai and analyze the risk factors for ARC to be better prepared for the increasing burden of cataracts as a significant cause of visual impairment worldwide.

**Methods:**

From March to June 2010, a population-based, cross-sectional study was conducted in a community selected by stratified cluster sampling in the Jingan district of Shanghai. Residents aged 40 and older were recruited and investigated by questionnaires and ophthalmic examination. Univariate and multivariate logistic regression models were used to evaluate the association of these risk factors with any cataract.

**Results:**

A total of 2894 subjects aged 40 years and above were included in our study. Nine hundred forty-eight people (32.8%) were diagnosed with cataract, including 845 with bilateral cataracts (29.2%) and 292 with moderate and severe visual impairment (low vision, 10.1%). There were significant differences in low vision among different age groups and gender (Χ^2^_age_ = 84.420, *P*_age_ < 0.001, Χ^2^_gender_ = 7.696, *P*_gender_ = 0.021). For any cataract, we found age (OR = 1.107, 95% CI: 1.094–1.120) and refractive error (OR = 1.352, 95% CI: 1.127–1.622) were independent risk factors.

**Conclusion:**

The prevalence of cataract is estimated to be nearly one-third of the sample, increasing with age. We provided further evidence that age and refractive error are independent cataract risk factors.

## 1. Introduction

The crystalline lens is a vital part of the intraocular refractive system to focus objects on the retinal through an adjustable function. The opacity of the crystalline lens leads to cataract and visual impairment, especially in older adults. Based on the etiology, oxidative stress is the direct mechanism of lens opacity. Compared to the general population, the physical and mental health and quality of life of people with cataract are more likely to be affected [1–3]. Cataract surgery is a kind of effective way to deal with cataract, but it is acompanied with many surgical complications and expensive costs that present enormous social and economic problems to a society, especially in the remote and poor areas of developing countries [4, 5]. According to the report of the World Health Organization (WHO), cataract is the leading cause of visual impairment in the world, accounting for most blindness (51%) [6]. The prevalence of any cataract in Sweden was 31.5% [7], in Russia was 44.6% [8], and in Myanmar was 40.39% [9]. Furthermore, China has entered an aging society, and Shanghai is the city with the highest degree of aging in China. It is estimated that in 2010, the population aged 60 and over was 3.4697 million (15.07%) in Shanghai, based on the sixth national census statistics. As an increasingly aging population trend, the incidence of age-related cataract has increased significantly in China, posing challenges for health systems. For example, the number of people over 45 years affected by any cataracts is expected to be 240.83 million, and those diagnosed with ARC will be 187.26 million by 2050 [5]. Therefore, understanding the epidemiology of senile cataract is the first step in prevention and treatment and may help to inform policymakers and healthcare providers better and be prepared for the increasing burden of cataracts.

Previous studies have been reporting a wide range of possible risk factors for age-related cataract (ARC), including increasing age, ultraviolet B exposure, smoking, drinking, estrogen, steroid hormone, antioxidants, diabetes, hypertension, and body mass index [1, 10, 11]. This report aims to be an exploration of the relationship between ARC and potential risk factors in an urban population in China.

## 2. Methods

### 2.1. Study Population

Jingan district is located in the center of Shanghai, covering an area of 7.62 square kilometers. In the sixth national census, the permanent population of the district in 2010 was 246788. Residents ≥40 years old in a street community in Jingan district were enrolled in this study. The study was fully conformed to the Declaration of Helsinki and approved by the Ethics Committee of Eye and ENT Hospital of Fudan University (Shanghai, China). All the participants were informed of the purpose and contents of the investigation in detail and signed the informed consent form before enrollment in the study. Ophthalmic examination included best corrected visual acuity (BCVA) and external and anterior segment examination with the slit lamp biomicroscope. The study workers performed a questionnaire interview for each participant concerning demographic details, education level, life habits, working conditions, and medical history.

### 2.2. Diagnostic Criteria

The Lens Opacity Classification System III ≥2 in either eye was defined as cataract. [12] We defined any cataract as meeting the criteria mentioned above and intraocular lens or aphakia eyes if the subject had an ARC surgery history in either eye, excluding low vision caused by other eye diseases. Patients with unilateral or bilateral cataracts were recorded as cataract patients. Visual acuity was categorized using the WHO criteria as follows: normal vision (BVCA ≥20/63), bilateral low vision (BCVA <20/63-≥20/400 in the better eye), and bilateral blindness (BCVA <20/400 in the better eye). [3].

### 2.3. Statistical Analysis

Statistical analysis was carried out using the SPSS version17.0 (IBM/SPSS, Inc., Chicago IL). The *T*-test and chi-square tests were used to analyze the univariate association of each risk factor with cataract and multivariable logistic regression with cataract as the dependent variable to access the independent associations for each risk factor. Odds ratios (OR) and 95% confidence intervals (95% CI) were presented, and differences were considered statistically significant when *P* < 0.05.

## 3. Results

### 3.1. Baseline Characteristics of the Subjects

Table 1 shows the baseline characteristics of the participants in this study. A total of 2894 people with an average of 62.9 ± 10.8 years ranging from 40 to 98 years old, were recruited, of which 1056 (36.5%) participated in the study were male and 1838 (63.5%) were female. Only a small number of elder people used computers (31.3%) and air conditioners (17%). On the contrary, most people have the habit of watching television (95.7%) every day with different watching times. With aging, hypertension occurred in most elderly people (38.1%), although most of the included subjects were not smoking (83.7%) and did not consume an alcoholic drink (86.1%).

### 3.2. Epidemiological Characteristics of Cataract

The prevalence of cataract by age and gender groups is shown in Table 2. Among the 2894 individuals who enrolled in the study, 948 (32.8%) were diagnosed with cataract, including 845 adults (29.2%) with binocular cataract and 103 adults (3.6%, right/left, 57/46) with monocular cataract. The prevalence of any cataract ranged from 5% in people aged 40–49 to 71.7% in those older than 80. The rate of cataract in adults 70–79 years old (62.9%) is two times and 12 times of that in adults 60–69 years old (29.9%) and 40–49 years old (5.0%), respectively. In addition, this study showed a significant difference in the prevalence of cataract among different age groups (Χ^2^ = 629.5, *P* < 0.001). The ratio of prevalence based on gender is male: female (350/598, 33.1%/32.5%) with no significant difference (Χ^2^ = 0.113, *P* = 0.737).

### 3.3. The Distribution of Visual Acuity

The bilateral visual acuity of 2884 people was obtained owing to 10 people refusing to exam (Table 3). The low vision rate ranged from 6.7% in people aged 40–49 to 27.6% in those older than 80. The ratio of low vision based on gender is male: female (88/204, 8.3%/11.1%). In addition, there were significant differences in low vision among different age groups and gender (Χ_age_^2^ = 84.420, *P*_age_ < 0.001, Χ_gender_^2^ = 7.696, *P*_gender_ = 0.021).

### 3.4. Risk Factors for Cataract

The risk factors for cataract were evaluated by univariate and multivariable analysis (Table 4). Using univariate analysis, age, marriage, hypertension, diabetes, and refractive error were risk factors of cataract, while the only refractive error was the risk factors of cataract (OR = 1.346, 95% CI: 1.124–1.612) after the age-adjust analysis. In multivariable regression model, only age (OR = 1.107, 95% CI: 1.094–1.120) and ametropia (OR = 1.352, 95% CI = 1.127–1.622) were independent risk factors of cataract. The multivariable analysis showed that people who had a history of ocular trauma (OR = 1.275) and wore contact lenses (OR = 5.664) had a higher risk of cataract than those who did not, although there was no significant difference.

## 4. Discussion

Our study provided new population-based data on the risk of cataract in urban residents aged 40 years and above with a large sample size. The prevalence of cataract aged 40 years old and over found in the Jingan district of Shanghai is 948 (32.8%) by investigating 2894 subjects, which was supported by Huang et al.'s study showing that the prevalence of cataract in the elderly is 39.86% in the Beixinjing area of Shanghai. [13] A systematic review projected that the prevalence (45–89 years of age) affected by any cataract in the whole of China is to increase from 22.78% (95% CI = 18.98–27.03) to 33.34% (95% CI = 28.53–38.40) between the years 2020 and 2050 [5]. However, the data from our study in urban Shanghai were much higher, probably due to the aggravated aging process and advances in diagnostic and geographical distribution [5]. In addition, the prevalence of cataract in Zheng et al.'s study with people older than 60 years in urban areas of Shanghai was 46.8%, similar to our present study (43.9%) [14].

As we know today, cataracts can be divided into three major types by cause, including age-related cataract, congenital cataract, and cataracts secondary to other causes. Notably, the most common type in adults is age-related cataract. In terms of visual acuity, we found that the number of people with normal vision significantly decreased gradually in this study, showing that older people tend to develop severe visual symptoms. There have been numerical studies to prove that increasing age is related to lens pathology as a risk factor, which is consistent with our study, suggesting the natural aging of the lens and the long-term exposure to potential risk factors [1, 15, 16]. Pathologically, oxidative stress is the direct mechanism of lens opacity. It has been found that the antioxidants and antioxidant enzymes in the eyes will be significantly reduced after 40 years old, resulting in the inability to protect the eyes effectively. Apart from that, the decreased protective pigment 3-hydroxycaninuric acid in the elderly eyes will be converted into phototoxic yellow uric acid, which may harm the lens [17].

Although male (33.1%) and females (32.5%) have similar prevalence of cataract, the less normal vision (89.4%) and more low vision (11.1%) are found in women, compared with men (91.4%, 8.3%) (Χ^2^ = 7.696, *P* = 0.021). We interpret these to mean that females tend to develop more visual significantly cataract. However, there was no significance between gender and cataract after multivariable analysis. The relationship between females and cataracts has been investigated by previous studies [5, 7, 18], while the mechanism behind sex disparity in cataracts remains to be elucidated. Presumably, a decrease in estrogen at menopause may be related to an increased risk of cataract in women due to the withdrawal effect rather than the concentration of estrogen [16]. However, long-term postmenopausal hormone therapy in women may increase their risk of cataract with type 2 diabetes [19] and cataract extraction [20]. Therefore, the evidence of the potential protective effect of hormone therapy against harmful oxidative stress will be the focus of future studies [5, 21].

Refractive errors are defined as common optical aberration determined by the cornea focusing power, lens, and ocular axial length, resulting from a complex interaction of lifestyle and genetic factors [22]. The mechanisms of refractive error pathogenesis remain to be investigated. The main mechanism can be divided into at least two sets: first, including all factors that alter refractive power; second, central neuro system-related, including circadian rhythm control [22, 23]. A cross-sectional study in Singapore showed that myopia (<−0.5D) was closely related to increased incidence of nuclear cataract (OR = 4.99) and posterior subcapsular cataract (OR = 1.34) [24]. And cataract surgery is also made more difficult in patients who have previously underwent corneal refractive laser surgery, such as intraocular lens power calculation [25, 26]. A recent meta-analysis showed a strong association with nuclear and posterior subcapsular cataract for any myopia, while cortical cataract tends to develop more in emmetropes and hyperopes than myopes [27, 28]. Our study also found refractive error as an independent risk factor for cataract (OR = 1.346, 95% CI: 1.124–1.612), consistent with previous studies. Myopia, one kind of ametropia, is well known as a strong factor in secondary cataract [1]. As previously described, a higher level of oxidative stress and byproducts of lipid peroxidation will occur in the myopia eye, possibly increasing cataract formation [27]. The longer axial length is proposed to be associated with the early cataract formation, which may be attributed to the weak diffusion of nutrients from the posterior chamber to the lens, but the lens is still in aqueous humor [27]. Instead, the development of cataract, in turn, can lead to a refractive error of the eyes, especially the nuclear cataract. Therefore, the relationship between nuclear cataract and myopia must be interpreted cautiously.

Cataract has been reported to be associated with lower education in the population of Korean [29], American [10, 30, 31], Chinese [32], Singapore [33], Myanmar [9], and Russian [8]. In the present study, the results of univariate regression analysis showed that those completing secondary education and above had a lower risk of cataract than those who were illiterate or completed primary school only. After adjusting for age, the protective factors only occur in people with a college education (OR = 0.722, 95% CI: 0.537–0.972). However, there was no apparent association between education level and cataract when the multivariable analysis was used. The mechanisms of education level relationship underlying this effect remain unknown. As a kind of socio-economic status, education level may reflect the discrepancy of lifestyle and environmental exposure, including ocular ultraviolet B exposure, health status, disease, and nutrition [15]. In addition, malnutrition has been proven as an independent risk factor for cataract [34]. For example, the proportion of antioxidant-rich vegetables in the diet of people with low education levels was significantly lower than that of people with high education levels, and the intake of antioxidants can significantly reduce the risk of cataract [35, 36].

Contrary to our expectation, the age-adjusted OR of cataract was found lowest in people who used computers every day for 3–8 hours. The educational level and occupation of the majority of the elderly in this study can explain this phenomenon because the educational level of people who used computers every day for 3–8 hours was higher than those who did not.

Increased smoking or alcohol consumption has also been linked to an increased risk of cataract [1, 11]. However, smoking or drinking was not significant after adjusting for age and multivariable analysis, which may be attributed to the misclassification by not recording past and current history [9].

There are some limitations to this study. Firstly, cataract patients who had taken measures to avoid possible risk factors after diagnosis were also included in the survey, which may lead to a decrease in the incidence of potential risk factors for cataract and even the opposite results of previous studies. Secondly, the subtypes of cataract were not recorded in this study, which may be one of the sources of negative results. However, the advantage of our study was the relatively large study population size and many variables to provide new evidence for the research of prevalence and cataract-related factors.

With the rapid growth of the aging population, cataracts remain the second largest cause of blindness worldwide and have a wide-ranging influence on social, economic, and health systems. Therefore, epidemiologically relevant research can help to inform policymakers and healthcare providers better and prepare for the increasing burden of cataracts.

## 5. Conclusion

In conclusion, our study investigated the prevalence of age-related cataract in the Jingan district of Shanghai and analyzed the risk factors for cataract. We proved that cataract is strongly associated with increasing age and refractive error.

## Figures and Tables

**Table 1:** Baseline characteristics of participants (*N* = 2894).

Variable		*N* = 2894	% (95% CI)
Gender	Male	1056	36.5 (34.7–38.2)
	Female	1838	63.5 (61.8–65.3)

Ethnic	Han	2891	99.9 (99.8–100.0)
	Others	3	0.1 (0.0–0.2)

Marital status	Married	2275	78.6 (77.1–80.1)
	Divorced	95	3.3 (2.6–3.9)
	Widowed	404	14.0 (12.7–15.2)
	Never married	120	4.1 (3.4–4.9)

Education	None/primary school only	601	20.8 (19.3–22.2)
	Secondary school	1872	64.7 (62.9–66.4)
	University	421	14.5 (13.3–15.8)

Smoking	Yes	472	16.3 (15.0–17.7)
	No	2421	83.7 (82.3–85.0)

Drinking	Yes	402	13.9 (12.6–15.2)
	No	2491	86.1 (84.8–87.3)

Time of using computer	No	1987	68.7 (67.0–70.3)
	1∼2 h/d	516	17.8 (16.4–19.2)
	3∼8 h/d	358	12.4 (11.2–13.6)
	>8 h/d	33	1.1 (0.8–1.5)

Time of using television	No	123	4.3 (3.5–5.0)
	1∼2 h/d	1190	41.1 (39.3–42.9)
	3∼8 h/d	1518	52.5 (50.6–54.3)
	>8 h/d	63	2.2 (1.6–2.7)

Time to use air conditioners	No	2401	83.0 (81.6–84.3)
	1∼2 h/d	275	9.5 (8.4–10.6)
	3∼8 h/d	188	6.5 (5.6–7.4)
	>8 h/d	30	1.0 (0.7–1.4)

Hypertension	Yes	1104	38.1 (36.4–39.9)
	No	1789	61.8 (60.0–63.6)

Diabetes	Yes	386	13.3 (12.1–14.6)
	No	2507	86.6 (85.4–87.9)

Infectious diseases	Yes	1	0.0 (0.0–0.1)
	No	2892	99.9 (99.8–100.0)

Refractive error	Yes	1398	48.3 (46.5–50.1)
	No	1495	51.7 (49.8–53.5)

Ocular trauma	Yes	49	1.7 (1.2–2.2)
	No	2844	98.3 (97.8–98.7)

Contact lenses	Yes	4	0.1 (0.0–0.3)
	No	2889	99.8 (99.7–100.0)

*N* = number; CI = confidence interval.

**Table 2:** Prevalence of cataract stratified by age and gender in the Jingan district of Shanghai in 2010.

Groups	No. of participants	Proportion%	Cataract	
			No.	% (95% CI)
*Age (years)*				
40–49	241	8.3	12	5.0 (2.2–7.7)
50–59	1038	35.9	160	15.4 (13.2–17.6)
60–69	777	26.8	232	29.9 (26.6–33.1)
70–79	598	20.7	372	62.2 (58.3–66.1)
≥80	240	8.3	172	71.7 (66.0–77.4)
			*P* < 0.001	

*Gender*				
Male	1056	36.5	350	33.1 (30.3–36.0)
Female	1838	63.5	598	32.5 (30.4–34.7)
			*P* = 0.737	
Total	2894	100	948	32.8 (31.0–34.5)

No. = number; CI = confidence interval.

**Table 3:** Age- and gender-specific prevalence of bilateral visual impairment using the definition of the World Health Organization in 2010.

Groups	No. of participants	Low vision		Blindness		Low vision and blindness combined	
		No.	% (95% CI)	No.	% (95% CI)	No.	% (95% CI)
*Age (years)*							
40 ∼ 49	240	16	6.7 (3.5–9.8)	—	—	16	6.7 (3.5–9.8)
50 ∼ 59	1035	77	7.4 (5.8–9.0)	6	0.6(0.1–1.0)	83	8.0 (6.4–9.7)
60 ∼ 69	775	57	7.4 (5.5–9.2)	4	0.5 (0.0–1.0)	61	7.8 (6.0–9.8)
70 ∼ 79	595	76	12.8 (10.1–15.5)	4	0.6 (0.0–1.3)	80	13.4 (10.7–16.2)
≥80	239	66	27.6 (21.9–33.3)	1	0.4 (−0.4–1.2)	67	28.0 (22.3–33.7)
		*P* < 0.001		*P* = 0.980^*∗*^		*P* < 0.001	
Male	1054	88	8.3 (6.7–10.0)	3	0.28 (0–0.6)	91	8.6 (6.9–10.3)
Female	1830	204	11.1 (9.7–12.6)	12	0.7 (0.3–1.0)	216	11.8 (10.3–13.3)
		*P* = 0.021		*P* = 0.182		*P* = 0.008	
Total	2884	292	10.1 (9.0–11.2)	15	0.5 (0.3–0.8)	307	10.6 (9.5–11.8)

No. = number; CI = confidence interval; — = data not available. ^*∗*^Fisher's exact test.

**Table 4:** Univariate and multivariable association analysis of risk factors for cataract.

Risk factors		Univariate analysis		After adjusting for age		Multivariate analysis		
		OR	95% CI	OR	95% CI	OR	95% CI	
Age		**1.116**	**1.106–1.126**			**1.107**	**1.094–1.120**	
Gender (female)		0.973	0.823–1.143	1.130	0.940–1.358	1.121	0.891–1.411	
Marital status	Never married	1		1		1		
	Married	0.925	0.620–1.382	0.796	0.509–1.246	0.845	0.537–1.330	
	Divorced	0.745	0.405–1.373	0.819	0.416–1.612	0.858	0.432–1.706	
	Widowed	**3.522**	**2.272–5.459**	0.976	0.529–1.610	0.949	0.568–1.583	

Education	Primary school or illiterate	1		1		1		
	Secondary school	**0.275**	**0.227–0.333**	0.835	0.659–1.057	0.901	0.703–1.154	
	University/college	**0.363**	**0.279–0.471**	**0.722**	**0.537–0.972**	0.850	0.613–1.179	

Time of using computer	No	1		1		1		
	1∼2 h/d	**0.485**	**0.388–0.606**	0.921	0.718–1.181	0.987	0.761–1.280	
	3∼8 h/d	**0.269**	**0.198–0.366**	**0.592**	**0.425–0.825**	**0.617**	**0.438–0.869**	
	>8 h/d	**0.352**	**0.145–0.856**	0.938	0.375–2.349	1.050	0.411–2.682	

Time of using television	No	1		1		1		
	1∼2 h/d	0.901	0.612–1.325	0.998	0.631–1.577	1.024	0.641–1.636	
	3∼8 h/d	0.794	0.542–1.164	0.971	0.617–1.528	1.017	0.639–1.619	
	>8 h/d	0.748	0.390–1.436	0.870	0.410–1.847	0.857	0.399–1.841	

Time of using air conditioners	No	1		1		1		
	1∼2 h/d	**0.554**	**0.413–0.743**	0.782	0.560–1.090	0.789	0.562–1.108	
	3∼8 h/d	**0.504**	**0.352–0.722**	0.849	0.561–1.266	0.864	0.576–1.295
	>8 h/d	0.568	0.243–1.328	0.869	0.331–2.282	0.858	0.324–2.276

Hypertension		**1.596**	**1.363–1.870**	0.975	0.810–1.172	0.942	0.779–1.139
Diabetes		**1.793**	**1.442–2.229**	1.211	0.943–1.556	1.183	0.915–1.530
Smoking		**0.754**	**0.606–0.938**	1.089	0.850–1.394	1.164	0.866–1.566
Drinking		0.809	0.640–1.019	0.966	0.741–1.257	0.971	0.720–1.309
Refractive error		**1.944**	**1.660–2.276**	**1.346**	**1.124–1.612**	**1.352**	**1.127–1.622**
Ocular trauma		1.092	0.603–1.976	1.367	0.711–2.628	1.275	0.659–2.469
Contact lenses		0.684	0.071–6.580	4.394	0.452–42.754	5.664	0.555–57.773

OR: odds ratio; the values in bold indicate that the *P* value is less than 0.05.

## Data Availability

The datasets used or analyzed during the current study are not publicly available due to our study containing individual person's data but are available partially from the corresponding author with reasonable request.

## Abbreviations

ARC: Age-related cataract
BCVA: Best corrected visual acuity
CI: Confidence intervals
OR: Odd ratios
SPSS: The Statistical Package for Social Sciences
WHO: World Health Organization.
